# Role of Microorganisms Present in Dairy Fermented Products in Health and Disease

**DOI:** 10.1155/2015/204173

**Published:** 2015-03-19

**Authors:** Clara G. de los Reyes-Gavilán, María Fernández, John Andrew Hudson, Riitta Korpela

**Affiliations:** ^1^Instituto de Productos Lácteos de Asturias, Consejo Superior de Investigaciones Científicas (IPLA-CSIC), Paseo Río Linares s/n, Villaviciosa, 33300 Asturias, Spain; ^2^Food Safety Programme, ESR-Christchurch Science Centre, Christchurch 8540, New Zealand; ^3^Food and Environment Safety, The Food and Environment Research Agency, Sand Hutton, York YO41 ILZ, UK; ^4^Medical Nutrition Physiology Group, Pharmacology, Institute of Biomedicine, University of Helsinki, 00014 Helsinki, Finland

Thousands of years ago humanity started agricultural practice and the domestication of cattle. Milk from farmed animals represented a good source of nutrients and liquid for hydration. The fermentation of milk provided a simple way to increase its shelf-life while improving its safety. From the initial accidental phenomenon of fermentation, humans learned to control these processes. Incorporating the controlled fermentation of milk in domestic practices of these primitive societies gave rise to a progressive diversification of dairy products, as influenced by habits of different ethnicities, geographical environments, and type of dairy farming. European-derived populations show lactase activity into adulthood, exhibiting selection for a lactase persistence haplotype [1]. The strong positive selective pressure exerted by animal husbandry practices resulted in the best studied phenomenon of gene-culture coevolution in the mutual human and animal symbiosis promoted by the advent of agriculture [2].

Therefore, fermented dairy products have been linked to the human nutrition and progress from ancient times. Nowadays they continue to be fundamental components of a balanced western diet. A huge variety of fermented dairy products are now available for consumers. Although a small proportion of these products are homemade, most of them are produced industrially; indeed, the dairy industry, particularly of fermented products, is economically important in many countries. Fermented products are generally populated by a diverse microbiota that impacts human health. Knowledge of microorganisms inhabiting underexplored natural fermented dairy products and their potential effects in human health, mechanisms underlying beneficial or detrimental effects of such microorganisms, and research in new safe alternative technologies to thermal processes constitute matters of current interest in food and health research. This special issue aimed to shed light on the role that microorganisms present in dairy fermented products play in human health and disease.

This special issue comprises reviews and experimental articles. Editors present first a general overview of the current state of the art. Although the special issue was opened to both beneficial and harmful microorganisms, contributions received focused on “good bugs.” Articles cover the following items: mechanisms of beneficial action of probiotics, food safety, and technological aspects of lactic acid bacteria and probiotics.

Relating mechanisms of probiotic action, the contributions address different aspects of microorganisms from the genus* Bifidobacterium*, a subdominant intestinal microbial group, some of whose strains have recognized probiotic effects. The beneficial action of probiotics is related, among others, to their capacity to colonize the host. V. Grimm and coworkers reviewed the mechanisms responsible for host colonization by bifidobacteria and factors involved. Two experimental articles dealing with the study of the mechanisms of probiotic-host interaction were presented. In one of them, N. Salazar et al. analyzed the capacity to modulate immune response and insulin-dependent glucose homeostasis by two exopolysaccharide-producing* Bifidobacterium* strains in a Wistar rat model. In the other, B. Sánchez et al. studied in an* in vitro* model the modification of the profile of immune mediators and proteins synthesized by the intestinal cell line HT29 in the presence of a strain of* Bifidobacterium breve,* concluding that the presence of bifidobacteria could favor innate immune response and reinforcement of the intestinal physical barrier.

M. J. Saez-Lara et al. reviewed the degree of scientific evidence in randomized human clinical trials for benefits associated with the use of lactic acid bacteria and bifidobacteria in the prevention and treatment of inflammatory bowel disease (Crohn's disease and ulcerative colitis) and other related diseases such as pouchitis and cholangitis.

Polyphenols are characterized by the presence of large multiples of phenol structural units that are synthesized by many vegetables as defense compounds. Many of them have antioxidant and other beneficial effects in human health. Finally the beneficial action of polyphenols greatly depends on the generation of bioactive compounds through their biotransformation by the intestinal microbiota. The addition of polyphenols to fermented dairy products deserves further research and development of technological applications. Two manuscripts reviewed the interaction of dietary polyphenols and the intestinal microbiota. M. Dueñas et al. analyzed the current knowledge on the modulation of the intestinal microbiota by these compounds from the point of view of the experimental approaches used. In contrast, L. Marín et al. explored the potentially beneficial action of dietary polyphenols as antiviral, antibacterial, and antiparasitic agents.

Five articles addressed technological aspects of beneficial microorganisms included in fermented dairy products, either by considering the behavior of probiotics during the manufacture process or by focusing towards the production of specific beneficial compounds by microorganisms during the elaboration of such products. J. M. Castro et al. reviewed aspects related to the potential of cheeses as probiotic carriers and some technological aspects related to the maintenance of the viability of probiotics in cheeses. An experimental contribution presented the development of a potential probiotic fresh cheese using two* Lactobacillus salivarius* strains isolated from human milk (N. Cárdenas et al.), a novel and interesting source for probiotics that is receiving considerable recent attention. The remaining three articles deal with the release of beneficial compounds by lactic acid bacteria and bifidobacteria in fermented dairy products. Thus, one article shows the capacity of lactic acid bacteria isolated from alpine traditional raw cow's milk cheese to produce *γ*-aminobutyric acid (E. Franciosi et al.) whereas another article (M. Gagnon et al.) analyzed the bioaccessibility of antioxidants during simulated digestion of milk that has been fermented by several strains of* Bifidobacterium longum* subsp.* longum*. In turn, M. A. Villar-Tajadura et al. demonstrated the ability to produce conjugated linoleic and *α*-linolenic acids by bifidobacteria from human milk in a milk-based medium. These articles open the possibility to use such strains for the development of fermented products with different functional properties.

Finally, three papers deal with the role of lactic acid bacteria in the safety of fermented dairy products. J. L. Arqués and colleagues review the antimicrobial activity against pathogens of lactic acid bacteria in dairy fermented products as well as in the gut after ingestion. In addition, two experimental articles address different aspects of safety in dairy products. Metagenomic analysis was used to characterize the presence of antibiotic resistance genes in the microbiota of a specific dairy fermented product,* Mozzarella di Bufala Campana* manufactured in Italy (C. Devirgiliis et al.). In turn, P. Carasi et al. determined safety aspects and antimicrobial properties of several strains of the species* Lactobacillus kefiri,* one of the most predominant microorganisms present in kefir-fermented milk.

This editorial presents a brief summary of the topics discussed in the articles published in this special issue. We hope readers will find useful information on the topics discussed here.
